# Supplementary material for: Hot spot prediction in protein-protein interactions by an ensemble system
Source: BMC Syst Biol. 2018 Dec 31;12(Suppl 9):132. doi: 10.1186/s12918-018-0665-8 (PMC6311905; doi:10.1186/s12918-018-0665-8)
Supplement: Supplementary file 2 — All relevant MATLAB code. A simple Matlab implement of our predictor. (ZIP 19,742 kb) [file 12918_2018_665_MOESM2_ESM.zip › Additional file 2/Read ASA/ASA Property/BID/1EBPC.txt]

 NetSurfP - Protein Surface Accessibility and Secondary Structure Predictions

  

|  |  |  |
| --- | --- | --- |
|  |  | NetSurfP - Protein Surface Accessibility and Secondary Structure Predictions  Technical University of Denmark |

  


---

```
# For publication of results, please cite:
# A generic method for assignment of reliability scores applied to solvent accessibility predictions.
# Bent Petersen, Thomas Nordahl Petersen, Pernille Andersen, Morten Nielsen and Claus Lundegaard
# BMC Structural Biology 2009, 9:51 doi:10.1186/1472-6807-9-51
#
# Column 1: Class assignment - B for buried or E for Exposed - Threshold: 25% exposure, but not based on RSA
# Column 2: Amino acid
# Column 3: Sequence name
# Column 4: Amino acid number
# Column 5: Relative Surface Accessibility - RSA
# Column 6: Absolute Surface Accessibility
# Column 7: Z-fit score for RSA prediction
# Column 8: Probability for Alpha-Helix
# Column 9: Probability for Beta-strand
# Column 10: Probability for Coil
E G  1EBPC                  1    0.897  70.555  -1.248   0.003   0.003   0.994
E G  1EBPC                  2    0.559  43.954  -2.513   0.004   0.138   0.858
E T  1EBPC                  3    0.507  70.279  -0.768   0.004   0.420   0.576
B Y  1EBPC                  4    0.231  49.365  -0.407   0.004   0.514   0.481
E S  1EBPC                  5    0.361  42.286   0.204   0.022   0.552   0.426
B C  1EBPC                  6    0.079  11.049  -0.286   0.023   0.655   0.322
B H  1EBPC                  7    0.273  49.731  -0.809   0.004   0.616   0.381
B F  1EBPC                  8    0.144  28.881  -0.326   0.004   0.514   0.481
B G  1EBPC                  9    0.196  15.425  -0.820   0.005   0.262   0.733
B P  1EBPC                 10    0.231  32.708  -0.840   0.004   0.197   0.799
B L  1EBPC                 11    0.091  16.589  -0.069   0.004   0.616   0.381
B T  1EBPC                 12    0.153  21.179  -0.103   0.002   0.816   0.182
B W  1EBPC                 13    0.063  15.055   0.009   0.011   0.918   0.071
B V  1EBPC                 14    0.096  14.724  -0.304   0.001   0.900   0.099
B C  1EBPC                 15    0.038   5.321  -0.997   0.001   0.900   0.099
E K  1EBPC                 16    0.351  72.242   0.964   0.003   0.718   0.279
B P  1EBPC                 17    0.239  33.971  -2.113   0.005   0.336   0.660
E Q  1EBPC                 18    0.571 101.998  -0.884   0.019   0.141   0.840
E G  1EBPC                 19    0.710  55.869  -2.873   0.018   0.047   0.935
E G  1EBPC                 20    0.923  72.664  -2.012   0.000   0.000   0.999

Explain the output.  Go back.
```

---
